# Exposure to Veterinary Antibiotics via Food Chain Disrupts Gut Microbiota and Drives Increased *Escherichia coli* Virulence and Drug Resistance in Young Adults

**DOI:** 10.3390/pathogens11091062

**Published:** 2022-09-18

**Authors:** Yehao Liu, Yifan Wu, Jie Wu, Xin Li, Lingling Yu, Ke Xie, Mingyi Zhang, Lingling Ren, Yanli Ji, Yuhui Li

**Affiliations:** 1 Departmen of Hygiene Inspection and Quarantine, School of Public Health, Anhui Medical University, Hefei 230032, China; 2 School of Biological Food and Environment, Hefei University, Hefei 230000, China

**Keywords:** veterinary antibiotics, preferred as veterinary antibiotics, gut microbiota, *Escherichia coli*, virulence, transcriptome analysis

## Abstract

Exposure to veterinary antibiotics (VAs) and preferred as veterinary antibiotics (PVAs) via the food chain is unavoidable for their extensive use not only for treating bacterial infections, but also for use as growth promoters in livestock and aquaculture. One of the consequences is the disturbance of gut microbiota. However, its impact on the virulence and drug resistance of opportunistic pathogens is still unclear. In this study, a total of 26 antibiotics were detected in the urine of 300 young undergraduates in Anhui Province. We found that excessive intake of milk was positively correlated to high levels of VAs and PVAs. It led to the dysbiosis of gut microbiota characterized by high abundance of Bacteroidetes and Proteobacteria. The increase in Proteobacteria was mainly due to a single operational taxonomic unit (OTU) of *Escherichia coli* (*E. coli*). We isolated several *E. coli* strains from participants and compared their drug resistance and virulence using PCR assay and virulence-related assays. We observed that exposure to high levels of VAs and PVAs induced more resistant genes and drove *E. coli* strain to become more virulent. At last, we conducted transcriptome analysis to investigate the molecular mechanism of virulent and drug-resistant regulators in the highly virulent *E. coli* strain. We noted that there were multiple pathways involved in the drug resistance and virulence of the highly virulent strain. Our results demonstrated that participants with high-level VAs and PVAs exposure have a disrupted gut microbiota following the appearance of highly drug-resistant and virulent *E. coli* and, therefore may be at elevated risk for long-term health complications.

## 1. Introduction

The human microbiota is a complex microbial community which plays an important role in metabolic regulation, food digestion, synthesis of new elements, intestinal barrier protection, etc. [1,2]. However, the balance of gut microbiota can be easily broken by some factors, such as drug, dietary, etc. [3,4,5]. The disrupted gut microbiota can increase the risk for several health complications, including decreased resistance against bacterial pathogens, predisposition to autoimmune and allergic diseases, and some chronic diseases [6,7,8]. Since VAs and PVAs are widely used in livestock and aquaculture around the world [9,10], exposure to them is unavoidable. However, their effects on the gut microbiota, especially on the characteristics of opportunistic pathogens, are not well known. 

The disrupted gut microbiota is often linked to highly elevated levels of Proteobacteria species, particularly *E. coli* [11,12]. *E. coli* is a Gram-negative bacterium that is the causative agent of many infective diseases, including diarrhea, synovitis, osteomyelitis, etc. [13,14]. Being part of the endogenous microbiota, multi-drug-resistant (MDR) *E. coli* strains can be induced as a consequence of extensive use of antibiotics [15]. The increasing prevalence of MDR and the virulent characteristics of *E. coli* need to be better understood, especially at the genetic bases, because it can help us to identify the contributing factors to the virulence and resistance of the isolated MDR strain.

This study used pyrosequencing of the 16S rRNA gene to explore the disrupted gut microbiota in young adults with antibiotic exposure via the food chain. Moreover, MDR *E. coli* was isolated from feces to determine pathogenic potential. To identify the drug-resistant and virulent regulators and global gene expression profile of the isolate, transcriptome analysis was conducted and compared to the transcriptome profile of reference strain *E. coli* ATCC 25922. Results from our study demonstrated the unique virulence and gene expression profile of MDR *E. coli* of human origin.

## 2. Materials and Methods

### 2.1. Antibiotic Analysis of Urine Samples

A total of 306 undergraduates were enrolled from Anhui medical university, and 300 undergraduates completed the study between December 2020 and November 2021. Figure S1 shows the survey process for the present study. Baseline data for the participants are shown in Table S1. Urine and stool samples were collected from these participants. The first morning urine samples were collected by participants themselves, immediately transported to the laboratory, and frozen at −20 °C for analysis. A total of 45 antibiotics from 9 categories, including human antibiotics (HAs), VAs, and PVAs, were chosen as the target antibiotics in this study. The pretreatment and analytical procedures of urine samples were described in our previously published method [16]. Briefly, after thawing and centrifuging, urine samples were mixed with internal standard, purified by solid-phase extraction, and analyzed by a liquid chromatography-triple quadrupole tandem mass spectrometry (LC–QqQ–MS/MS).

### 2.2. Dietary Investigation

To investigate the relationship between food consumption and antibiotic level in urine samples, simplified food-frequency questionnaire (FFQ) was used in this study, which was described by a previous report [17]. Briefly, the daily dietary intake was determined by FFQ, which contained 115 types of food commonly consumed in China. All these foods were classified into animal-derived foods and vegetable-derived foods. Dietary intake investigation included whether the food was consumed, consumption frequency, and amount of food consumption at each time.

### 2.3. Collection of Stool Samples and Extraction of Bacterial Genomic DNA for High-Throughout Sequencing

All stool samples were collected using sterile cups and stored at −20 °C until analysis except for *E. coli* isolation. Genomic DNA was extracted from stool samples using QIAGEN QIAamp Fast DNA Stool Mini kit (QIAGEN, Hilden, Germany) according to the manufacturer’s instruction. After that, DNA concentration was measured by Nanodrop 2000 spectrophotometer (Thermo Scientific, Waltham, MA, USA). The 341F and 806R primers were chosen to amplify bacterial 16S rRNA gene targeting the V3–V4 region. The PCR products were purified using the Gel Extraction Kit (CWbio, Beijing, China) according to the manufacturer’s instructions and quantified using Nanodrop 2000. Next, purified PCR products were mixed together in equal amounts and paired-end sequenced on the platform of Illumina Miseq (Illumina, San Diego, CA, USA). After quality checking, qualified raw reads were processed using Quantitative Insights Into Microbial Ecology (QIIME) software. Operational taxonomic units (OTUs) were delineated at 97% sequence similarity. Alpha and beta diversity indices were calculated using QIIME. Linear discriminant analysis effect size (LEfSe) analysis was performed with the online tool (https://huttenhower.sph.harvard.edu/galaxy, accessed on 18 February 2022).

### 2.4. E. coli Quantification in Stool Samples

The quantity of *E. coli* in stool samples was determined by quantitative PCR (qPCR) as described by Fazelahi et al. [18]. Briefly, extracted genomic DNA was used as template DNA, and a set of primers and a Taqman probe were designed targeting a conserved sequence of 23S rDNA of various species of *E. coli*. The sequences of primers are listed in Table S2. The qPCR mix contained 25 µL of 2× Premix Ex Taq^TM^ (Takara, China), 0.3 µM TaqMan probe, 0.6 µM of each primer, and 60 ng of template DNA, and water was added to make the final volume to 50 µL.

### 2.5. Isolation and Identification of E. coli from Stool Samples

Fresh stool sample was dissolved in sterile phosphate buffer (pH 7.0). After 2000 rpm centrifuging for 2 min, about 100 µL supernatant was streaked on Violet Red Bile agar and incubated at 37 °C overnight. The typical purple colonies were then streaked on Eosin Methylene Blue (EMB) agar plates and were incubated at 37 °C overnight. All microbial culture medium products were purchased from Qingdao Hi-tech Industrial Park Hope Bio-technology Co. Ltd. Suspected colonies of *E. coli* were picked up and identified by sequencing 16S rRNA gene.

### 2.6. The Virulence-Related Assays of the Isolates

The ability of biofilm formation was assessed by crystal violet staining method as previously described [19]. Briefly, the cells were collected after overnight growth and inoculated to Luria-Bertani (LB) broth. The final cell concentration was diluted to 1.0 × 10^5^ CFU.mL^−1^ and 200 µL of bacterial inoculum was added to each well of polystyrene microtiter plate for incubation at 37 °C. Prior to measuring, the wells were washed gently twice by deionized water to remove cells. After drying for 15 min, 100 µL of 1% crystal violet solution was added to each well to stain the biofilm for 30 min. After that, the wells were washed gently by tap water to remove unbounded crystal violet. About 100 µL of dissolving solution (water solution with 30% methanol and 10% acetic acid) was added to each well to dissolve crystal violet and the optical density (OD) was measured by a microplate reader at 570 nm wavelength.

The activity of endotoxin in culture’s supernatant was measured using Bioendo Limulus Amebocyte Lysate kit (LAL, Xiamen, China) according to the manufacture’s instruction. Briefly, endotoxin-free water, pipette tips, and tubes were used, and endotoxin of *E. coli* O111:B4 (Sigma-Aldrich Corporation) was used to construct the calibration curve. After successive dilutions, 5 concentrations (1 EU.mL^−1^, 0.5 EU.mL^−1^, 0.25 EU.mL^−1^, 0.1 EU.mL^−1^, and 0 EU.mL^−1^) were obtained to construct the calibration curve. The activity of endotoxin was determined according to the calibration curve.

To measure swimming motility of the isolates, overnight-grown cells were collected by centrifuge and washed twice by PBS. After that, cell concentration was adjusted to 1.0 × 10^8^ CFU.mL^−1^. About 5 µL of bacterial inoculum was stabbed on a semi-solid agar plate and incubated at 37 °C. The motility halos were obtained by measuring the diameter of the circular zone every six hours. Motility halos of the isolates were compared and statistically analyzed.

### 2.7. Detection of Drug-Resistant Genes in the Isolates by PCR Assay

PCR assay was conducted to detect antibiotic-resistant genes. Briefly, the cells were harvested from LB broth after overnight growth. Genomic DNA was extracted using Ezup Column Bacteria Genomic DNA Purification Kit (Sangon, Shanghai, China) according to the manufacture’s instruction. The primer sequences are listed in Table S2. PCR products were visualized via electrophoresis in 2% agarose gel with SYBR green staining and imaged on a UV trans-illuminator.

### 2.8. RNA Sequencing and Data Analysis

The analysis of RNA sequencing was performed as previously described [20]. Briefly, the cells were harvested at log phase. Total RNA was extracted from these cells using an Ultrapure RNA kit (CWbio, Beijing, China) and used to construct transcriptome libraries. The libraries were generated on Illumina HiSeq 2500 platform at Shanghai Personal Biotechnology Corporation. Raw reads were qualified by removing adapters, poly-N, and low-quality reads. All following analyses were based on these clean, high-quality reads. The level of gene expression was determined by the number of fragments per kilobase of the transcript sequence per million base pairs sequenced (FPKM). The analysis of the differentially expressed genes (DEGs) was conducted using DESeq R package.

Gene ontology (GO) enrichment analysis of the DEGs was performed via GOseq R packages. The role of DEGs in some pathways was consulted to the Kyoto Encyclopedia of Genes and Genomes (KEGG) database.

### 2.9. Validation of Gene Expression by Quantitative Real-Time PCR (Qpcr)

We conducted qPCR to amplify 5 DEGs to validate the result of RNA sequencing analysis. Briefly, total RNA of each sample was reversely transcribed by All-in-one 1st Strand cDNA Synthesis SuperMix Kit (Novoprotein, Beijing, China) according to the manufacture’s instruction. All qPCRs were conducted with NovoStart SYBR qPCR SuperMix Plus kit (Novoprotein, Beijing, China) in Roche Lightcycler 96 (Roche, Santa Clara, CA, USA), and primer sequences are listed in Table S1. The expression of each gene was verified by three independent qPCR reactions. The house-keeping gene, 16S rRNA, was selected as an internal standard. The relative expression of each gene was calculated using the 2^ΔΔ^Ct method.

### 2.10. Statistical Analysis

Data analysis was conducted using SPSS version 22.0. A two-tailed *t*-test was used to calculate the mean ± standard deviation. Statistical significance was considered at the *p* < 0.05 level. Three biological replicates were conducted for all experiments.

## 3. Results

### 3.1. High Intake of Milk Is Positively Correlated to the Level of Antibiotic Residues in Urine Samples

All participants did not administer any antibiotics in the past year. However, a total of 26 types of antibiotics were detected in their urine samples (Table 1). Of these antibiotics, 2 HAs, 8 VAs, and 16 PVAs were detected. Among the participants, one group had more types of antibiotics (26 types) with high concentration in urine samples; it was named high-level antibiotics group (HAG). Another group had less types of antibiotics (23 types) with low concentration in urine samples; it was named low-level antibiotics group (LAG). Interestingly, one VA and two PVAs were only detected in HAG.

**Figure 1:**
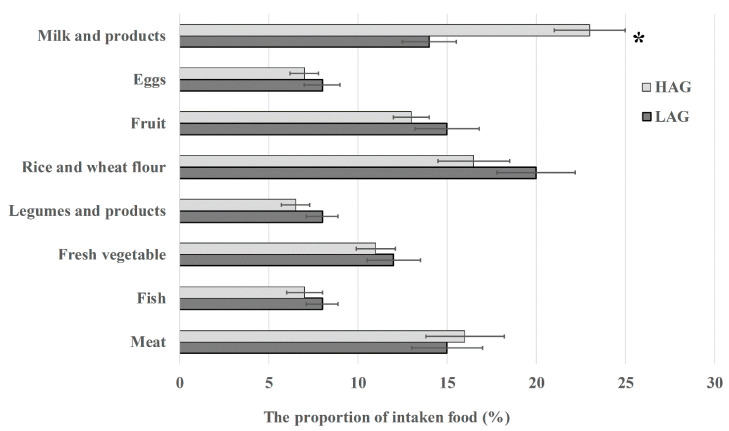
The proportion of different types of food consumed by LAG and HAG. * *p* < 0.05.

Since VAs and PVAs have been widely used in aquaculture and agriculture, they can enter into human bodies via the food chain. To investigate which food can explain the difference in antibiotic level, we compared the dietary composition between the two groups. Among the 149 items, along with the recipes commonly used in China, only milk intake was significantly higher in HAG than that in LAG (Figure 1). Combining the results of participants’ medical history and dietary habit, we deduced that massive intake of milk may contribute to the high level of antibiotic residues in urine samples.

### 3.2. The Structure of Gut Microbiota in HAG Is Totally Different from LAG

Even exposure to low-level antibiotic can alter the structure of gut microbiota. To investigate the difference in gut microbiota between the two groups, we generated a profile of bacterial community for each sample through PCR amplification of the 16S rRNA gene, followed by Illumina high-throughput sequencing. After quality checking from raw reads, we harvested qualified reads ranging from 40,159 to 43,319. According to the result of rarefaction curve (data not shown), high sampling coverage was achieved in all samples. These data demonstrated that sequencing depth was adequate to analyze gut microbiota. There were 2681 unique OTUs in HAG, 2215 unique OTUs in LAG, and 335 OTUs were shared by both groups.

**Figure 2:**
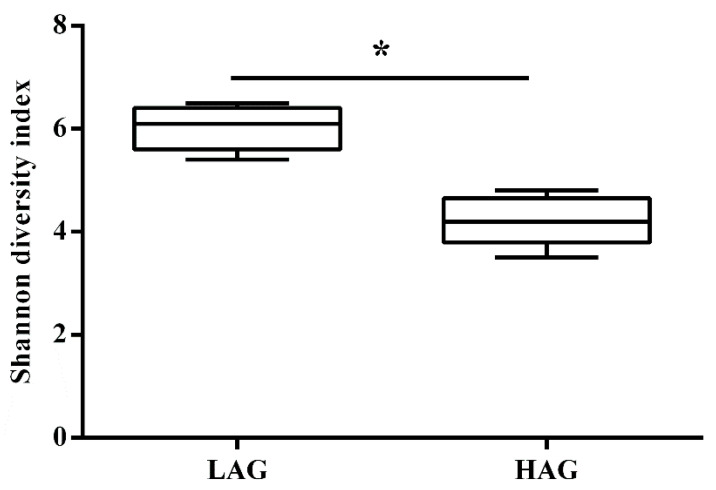
The comparison of Shannon diversity index between LAG and HAG. * *p* < 0.05.

**Figure 3:**
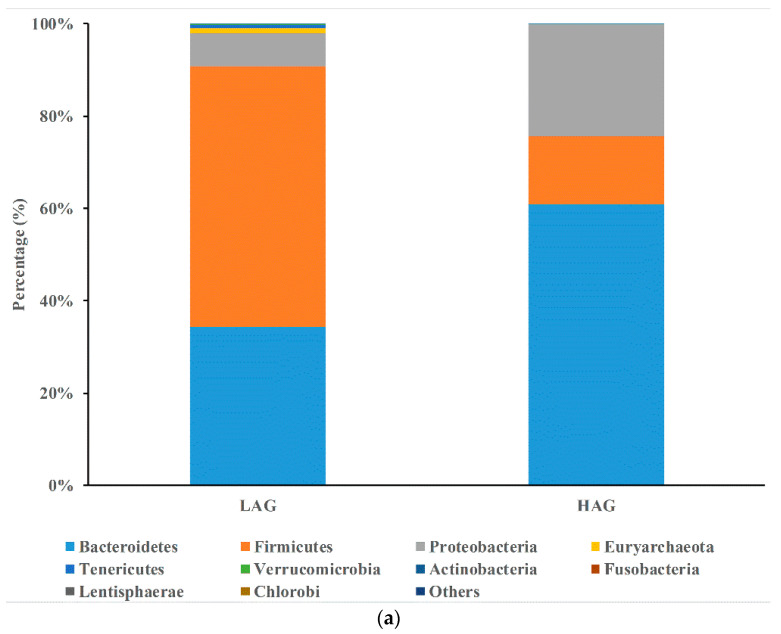

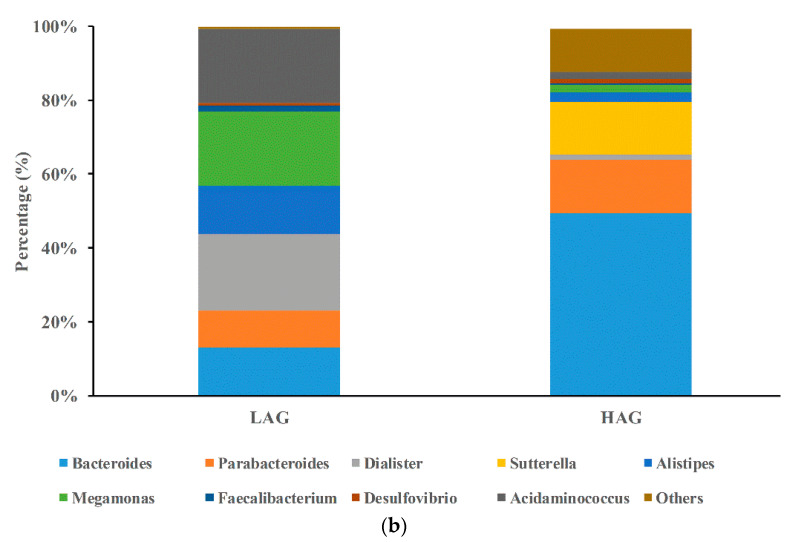
(**a**) The relative abundance of predominant bacteria at the phylum level in LAG and HAG. (**b**) The relative abundance of predominant bacteria at the genus level in LAG and HAG.

As reflected by within-sample diversity (α-diversity), we found that the structure of gut microbiota was totally different between the two groups; Shannon index of HAG was significantly lower than that of LAG (Figure 2). Compared at the phylum level (Figure 3a), HAG had a high proportion of Bacteroidetes (60.8%) and Proteobacteria (24.4%), while LAG had a high proportion of Firmicutes (56.6%) and low Bacteroidetes (34.2%). At the genus level (Figure 3b), HAG had a high proportion of *Bacteroides* (49.4%) and *Proabacteroides* (14.2%), whereas LAG had a high proportion of *Dialister* (20.8%) and *Alistipes* (13.0%).

**Figure 4:**
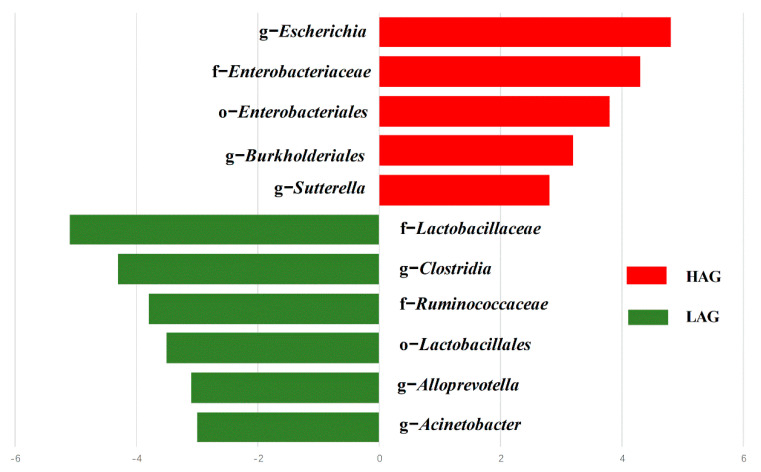
The most differentially enriched taxa between LAG and HAG which were identified via LDA score based on linear discriminant analysis effect size (LEfSe) analysis.

Next, LEfSe analysis was performed to find the specific bacterial taxa that explained the difference. As shown in Figure 4, several species at different taxonomic levels could explain the differences which were described by a linear discriminant analysis score of 2 or greater. We found an enrichment of *Sutterella*, *Enterobacteriaceae*, *Escherichia,* and *Enterobacteriales* in HAG, and most of the enriched taxa were belonged to Bacteroidetes and Proteobacteria, whereas several non-Bacteroidetes taxa, including *Lactobacillaceae*, *Lactobacillales*, *Ruminococcaceae*, *Clostridia*, *Acinetobacter*, and *Alloprevotella* were enriched in LAG.

### 3.3. HAG Has High abundance of E. coli

**Figure 5:**
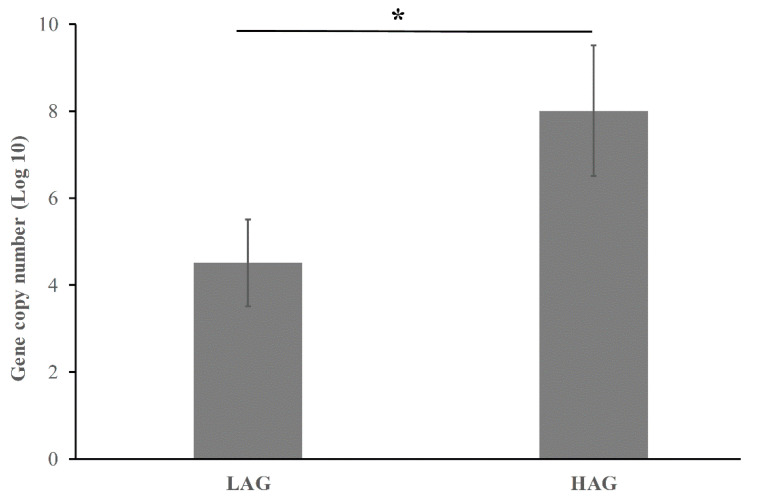
Quantification of total *E. coli* in fecal samples from LAG and HAG. Bars represent the log10 gene copy number. * *p* < 0.05.

*E. coli* is an important member of the normal gut microbiota in human bodies. It can act as a harmless gut commensal to intra- or extra-intestinal opportunistic pathogen [3]. Our result based on the analysis of gut microbiota showed that *E. coli* was enriched in HAG. To confirm this finding, a qPCR assay was conducted to quantify all strains of *E. coli* that existed in the stool samples. As shown in Figure 5, the abundance of *E. coli* was significantly higher in HAG than that in LAG. It was consistent with the result of LEfSe analysis.

### 3.4. E. coli Isolated from HAG Harbors More Resistant Genes

Antibiotic-resistant genes are involved in *E. coli*’s resistance to antibiotics, such as ampicillin, norfloxacin, etc. [21]. Since HAG received more exposure from VAs and PVAs, and had high abundance of *E. coli*, we supposed that *E. coli* isolated from HAG can induce more resistant genes. To confirm it, we performed a PCR assay to detect a total of 16 reported resistant genes among the isolated *E. coli* strains. We isolated a strain from HAG harboring 11 resistant genes (Table 2), including *cml*A gene from chloramphenicol, *tet*A gene from tetracycline, *OXY* and *ctx*-M1 genes from β-lactam, genes for coding multidrug efflux pumps (*mdt*l, *mdt*G, *mdt*F, and *mdt*b), and three genes from quinolone (*qnr*-s, *qnr*-B, and *qnr*-A). However, we only isolated a strain from LAG harboring six resistant genes. The result suggested that many resistant genes can be induced even by low-level VAs and PVAs exposure.

### 3.5. Virulence-Related Abilities of the Isolate from HAG Are Stronger Than the Isolate from LAG

Since *E.* coli has the ability to cause extra- or/and intra-intestinal human infection, a high-virulence isolate may increase the risk to its host’s health. The virulence-related abilities of the isolates were compared in vitro in this section.

**Figure 6:**
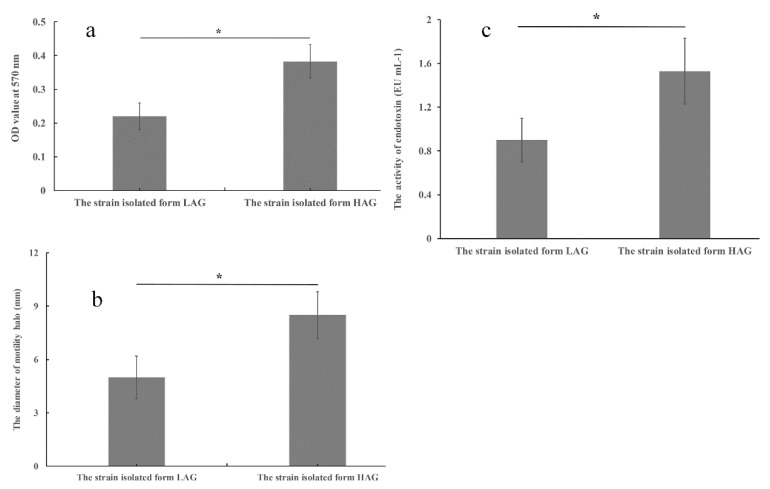
The comparison of virulence-related abilities of the two isolates. (**a**) The comparison of biofilm formation between the two *E. coli* isolates. (**b**) The comparison of swimming motility between the two *E. coli* isolates. (**c**) The comparison of endotoxin activity in culture’s supernatant. The cell population is adjusted to the same OD value before collecting supernatant by centrifuge. The result is shown in the form of mean value of three independent repeats. * *p* < 0.05.

Biofilm formation is a survival and protective strategy for pathogens, including *E. coli*. Bacterium within biofilm shows increased resistance to antibiotics and other chemical compounds [22]. As a result, microbial biofilm formation is regarded as an important virulence factor. To investigate whether there is any difference in biofilm formation between the isolates, crystal violet staining assay was performed. As shown in Figure 6a, the isolate from HAG showed a significant increase in OD value by 31.7% when compared to the isolate from LAG. The result suggested that the isolate from HAG has the high ability of biofilm formation.

Many species, including *E. coli*, use flagella to propel themselves to move toward food or away from noxious environments. This flagellum-mediated motility is called swimming motility, and this ability is also positively related to microbe’s virulence [23]. To explore whether there is any difference in swimming motility between the isolates, we performed swimming motility assay on semi-solid agar. As shown in Figure 6b, the isolate from HAG showed significant increase in swimming motility when compared to the isolate from LAG.

Exposure to antibiotics can accelerate the release of endotoxin, which is regarded as the important bacterial factor in the pathogenesis of Gram-negative septic syndrome [24]. To compare the difference in endotoxin release of the two isolates, we performed an endotoxin-releasing assay. As shown in Figure 6c, the isolate from HAG released more endotoxin than that of the isolate from LAG. The above data indicated that the isolate from HAG exhibited higher virulence than the isolate from LAG.

### 3.6. Transcriptome Analysis

To investigate the molecular mechanism of the regulators involved in virulence and drug resistance of the isolate from HAG, we performed transcriptome analysis. *E. coli* ATCC 25922 was chosen as the reference strain. Briefly, a total of 26,498,848 to 32,741,819 raw reads of 150 bp-paired ends were harvested. An average of 28,038,670 high-quality reads was obtained after removing adapters and low-quality reads. All these high-quality reads were mapped to the reference genome. The average mapped rate was 79.97% and 3923 genes were aligned to the database. 

The result of DEGs analysis revealed that many genes are involved in modulating virulence and drug resistance of the isolate. Compared to the reference strain, 186 genes showed twofold or higher differential expression. In detail, 98 genes were up-regulated while 88 genes were down-regulated. The full list of these genes can be found in Tables S3 and S4.

According to the result of KEGG pathway analysis, the 98 up-regulated genes were classified into three categories: the first category was involved in the resistance to several environmental factors, including heat shock protein, arsenate reductase, osmotically-inducible lipoprotein, arsenical resistance operon repressor, and cold-shock-like protein. The second category was involved in the virulence, including lipopolysaccharide core heptose(II)-phosphate phosphatase, biofilm regulator *Bss*R, *Hha* toxicity modulator *Tom*B, biofilm synthesis protein, and Colicin V production protein. The third category was involved in the cell structure, including arsenical pump membrane protein, transport protein *Tqs*A, inner membrane protein *Yeb*E, sodium/proton antiporter *Cha*A, periplasmic protein *Cpx*P, inner membrane protein *Yea*I, and inner membrane transport permease *Ybh*R.

**Figure 7:**
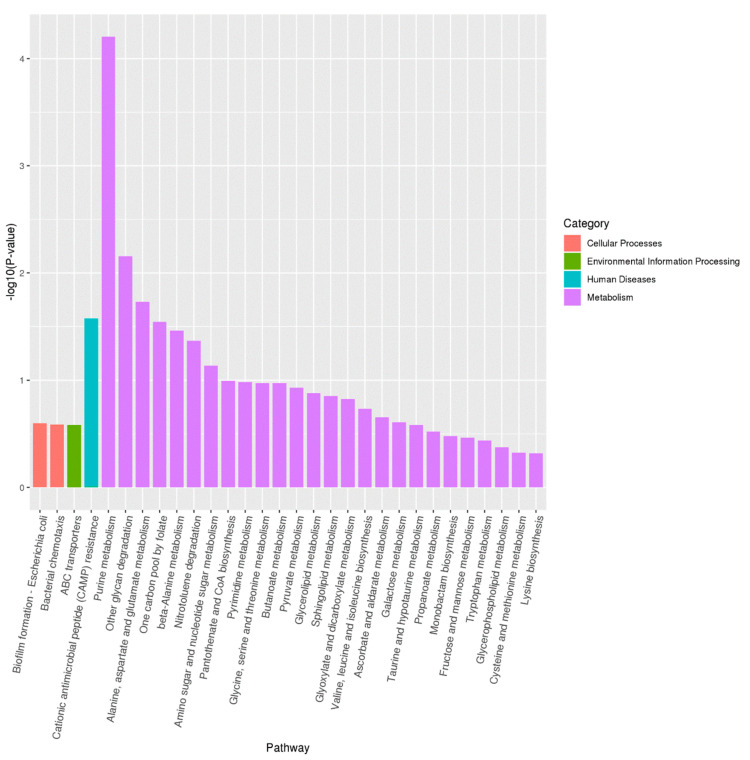
Histogram of the KEGG pathway enrichment annotations of the differentially expressed genes (DEGs) between the isolate from HAG and reference strain *E. coli* ATCC 25922. The x-axis shows functional pathways and the y-axis shows statistical significance (*p*-value).

The 88 down-regulated genes were classified into two categories: the first category was involved in the metabolism, including propionate kinase, lysine decarboxylase, and ethanolamine utilization protein. The second category was involved in cell structure, including inner membrane protein and inner membrane transport protein. Together, the result of KEGG pathway enrichment analysis showed that several pathways, including biofilm formation, bacterial chemotaxis, ABC transporters, cationic antimicrobial peptide (CAMP) resistance, and purine metabolism, were enriched in the isolate (Figure 7), indicating that these KEGG pathways played important roles in the virulence and drug resistance of the isolate.

**Figure 8:**
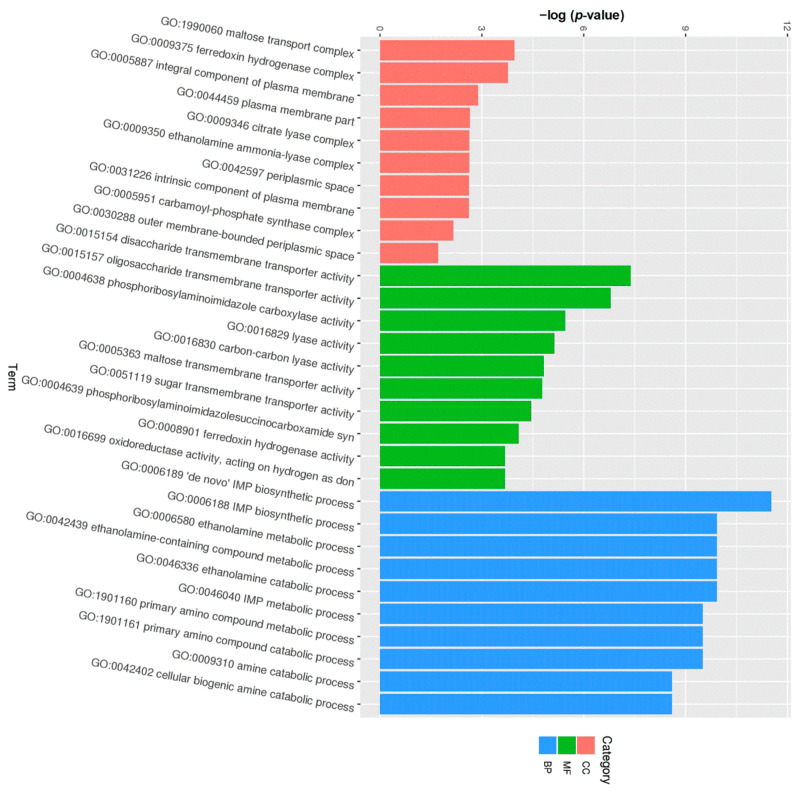
Histogram of GO functional enrichment annotations of the differentially expressed genes (DEGs) between the isolate from HAG and reference strain *E. coli* ATCC 25922. The x-axis shows top 10 terms in the three main categories, including cellular component (CC), biological process (BP), and molecular function (MF), and the y-axis shows statistical significance (*p*-value).

According to the result of GO classification, several GO terms were enriched in the isolate from HAG. As shown in Figure 8, in the category of cellular components, some DEGs were involved in maltose transport complex and integral component of plasma membrane. In the category of molecular functions, some DEGs were involved in the activities of disaccharide transmembrane transporter, oligosaccharide transmembrane transporter, and phosphoribosylaminoimidazole carboxylase. In the category of biological processes, some DEGs were involved in the biosynthetic process of hypoxanthine nucleotide and ethanolamine metabolism.

### 3.7. Validation of the Results of Transcriptome Analysis via qPCR

In order to validate the RNA sequencing data, we randomly chose five genes from the list of DEGs and conducted qPCR. We successfully amplified five DEGs and obtained a single band checked by agarose gel electrophoresis. Among the five genes, two genes were down-regulated and three genes were up-regulated. As shown in Figure S2, the expression pattern of these genes was consistent with the RNA-sequencing analysis, suggesting that the results of transcriptome analysis are reliable.

## 4. Discussion

Since both VAs and PVAs have been widely used in aquaculture and agriculture, antibiotic residues can enter into human bodies through animal-derived foods, such as meat, milk, and egg products [25,26]. Therefore, drug level in the urine of young adults without drug administration can reflect the long-term drug exposure via the food chain. In this study, we found that milk was the main source of antibiotic residues in urine. A high level of antibiotic residues caused the dysbiosis of gut microbiota characterized by high proportions of Bacteroidetes and Proteobacteria. High drug-resistant and virulent *E. coli* was enriched. It may cause an elevated risk for long-term health complications.

Contamination by antibiotics has been proved to generate multiple harmful effects on human health, even at low concentration [27,28]. In contrast to the antibiotic usage for medical purpose, which can be controlled strictly, exposure to VAs and PVAs is unavoidable and a long-term process [29]. In this study, we found that milk intake is positively correlated to the high level of antibiotic residues. Although antibiotic level in milk was not measured in our study, previous research reported that the levels of sulfonamides and quinolones in milk were from 16.28 mg kg^−1^ to 23.25 mg kg^−1^ among 10 provinces of China [30]. Milk products containing antibiotic residues are common around the world, which is reviewed by Sachi et al. [31]. Therefore, excessive intake of milk brings high risk of antibiotic exposure.

Antibiotic exposure can disrupt gut microbiota and increase the risk for several health complications, including obesity, increase in bacterial pathogens, etc. [32,33]. A previous study found that the diversity of gut microbiota is decreased after exposure to antibiotics, whereas a high diversity gut microbiota is observed in healthy control [34]. In our study, we also observed the disrupted gut microbiota in HAG with decreased diversity. The low Shannon index means that the balance of gut microbiota is broken and the abundance of some dominant taxa is decreased, while some rare taxa become more abundant [35]. In our study, a similar phenomenon is described by the comparison of relative abundance of predominant bacteria at the phylum and genus levels between the two groups, respectively. We found that the abundance of Bacteroidetes and Proteobacteria was significant higher in HAG than that in LAG. Our finding is consistent with other findings reporting that Bacteroidetes and Proteobacteria are more resistant to antibiotic exposure than other taxa [36,37]. The result of LEfSe analysis further demonstrated that *E. coli* was enriched in HAG. *E. coli* has been described in many reports for their high resistance to antibiotics [38]. As a result, it is reasonable to deduce that low diversity of gut microbiota and high abundance of Bacteroidetes and Proteobacteria are a biomarker of long-term exposure to VAs and PVAs via the food chain.

Besides the disrupted gut microbiota, exposure to antibiotic also can induce drug resistance in some bacterial taxa [39]. We found that *E. coli* induced more resistant genes in HAG than that in LAG. As a result, it led to the high abundance of drug-resistant *E. coli* in HAG, which was validated by qPCR. It has been reported that elevated abundance of *E. coli* is linked to increased inflammation during infection [40]. Although no illness was found in HAG, disrupted gut microbiota with elevated *E. coli* is a risk factor for long-term health complications, which has been reported in many studies [38,41].

Transcriptional analysis allows us to understand the molecular mechanism of drug-resistant and virulent regulators [42]. The possible function of DEGs can be deduced because the genes within the same pathway usually co-operate with each other to run their biological function [43]. It has been demonstrated that enhanced efflux can extrude drugs from inside the cell. As an example, drug extrusion is an effective resistance mechanism in *Salmonella* and *E. coli*, where efflux pump confers resistance to fluoroquinolone and tetracycline [44]. According to KEGG pathway analysis, genes involved in trans-membranes transporters were up-regulated. It can enhance drug resistance of the isolate. Interestingly, we observed that the genes involved in the resistance to several environmental factors were up-regulated too. These results indicate that, besides resistance to antibiotics, the isolate from HAG may have high resistance to environmental factors, such as temperature and osmotic pressure. We will validate it in future work. High drug resistance is often associated with reduced bacterial fitness. One of the biological costs is growth delay [45]. Most of the down-regulated genes in the isolate were involved in cell growth. Our finding is consistent with the report. Drug resistance and virulence are not two independent characteristics but, rather, a negative or positive relationship can be found between them [46]. We found that there was a positive relationship between drugresistance and virulence in the isolate. This relationship provides advantages to the isolate, allowing the isolate to survive in different niches with different selective pressures, such as antibiotics, etc.

## 5. Conclusions

In this study, we found a positive correlation between milk intake and the level of VAs and PVAs in participants’ urine. Exposure to VAs and PVAs caused a dysbiosis of gut microbiota, which was characterized by high proportions of Bacteroidetes and Proteobacteria. *E. coli* isolate exhibited high virulence and drug resistance. At last, we investigated the possible molecular mechanism of drug-resistant and virulent regulators in the isolate at the transcriptional level. However, there are several limitations in this research. First, dietary habits were not well surveyed, and antibiotic level in foods was not determined. Second, only *E. coli* was chosen for further study. Third, the drug-resistant and virulent regulators in the isolate were only studied at the transcriptional level.

## Figures and Tables

**Table 1:** Detected antibiotics in urine samples.

Antibiotic Type	Antibiotics	Healthy Group (ng/mL)	Patient Group (ng/mL)	*p*-Value
HAs	Levofloxacin	6.22	6.35	NS
	Clarithromycin	0.97	0.94	NS
VAs	Enrofloxacin	2.79	2.66	NS
	Sulfachloropyridazine	51.34	61.56	*
	Sulphaquinoxaline	1.24	2.67	*
	Sulfaclozine sodium monohydrate	13.44	51.54	*
	N4-acetylsulfamonomethoxine	4.35	4.55	NS
	Cefquinome sulfate	59.57	92.94	*
	Ceftiofur	0.00	1.55	*
	Cyadox	315.38	322.81	NS
PVAs	Lomefloxacin hydrochloride	5.53	8.96	*
	Ofloxacin	0.00	10.17	*
	Ciprofloxacin	13.48	14.22	NS
	Norfloxacin	2.43	5.26	*
	Trimethoprim	4.90	4.33	NS
	Sulfametoxydiazine	12.65	24.37	*
	Sulfamethoxazole	4.42	7.37	*
	Sulfadiazine	75.74	111.23	*
	Erythromycin	54.43	73.63	*
	Lincomycin hydrochloride	11.24	26.29	*
	Doxycycline hydrochloride	84.39	110.01	*
	Tetracycline hydrochloride	4.34	3.89	NS
	Cefotaxime sodium	22.19	30.23	*
	Amoxicillin trihydrate	62.12	229.51	*
	Penicillin-G sodium salt	133.06	421.33	*
	Penicillin V	0.00	4.23	*

**Note:** NS: not significant; *: *p* < 0.05; HAs: human antibiotics, VAs: veterinary antibiotics, PVAs: preferred as veterinary antibiotics.

**Table 2:** The list of resistant genes detected in the isolates.

Isolate’s Name	Antibiotic-Resistant Genes Detected in the Isolate
*E. coli* strain isolated from LAG	*cml*A, *tet*A, *mdt*F, *mdt*b, *ctx*-M1, *qnr*-A
*E. coli* strain isolated from HAG	*cml*A, *tet*A, *OXY*, *mdt*l, *mdt*G, *mdt*F, *mdt*b, *ctx*-M1, *qnr*-s, *qnr*-B, *qnr*-A

## Data Availability

The data presented in this study are available within the article.

## Supplementary Materials

The following supporting information can be downloaded at: https://www.mdpi.com/article/10.3390/pathogens11091062/s1, Figure S1: The follow-up diagram of the present study; Figure S2: Comparison of qPCR result with RNA-Seq; Table S1: Baseline characteristics of study participants; Table S2: Primers used in this study; Table S3: Up-regulated genes; Table S4: Down-regulated genes.
