# Supplementary material for: Exposure to Veterinary Antibiotics via Food Chain Disrupts Gut Microbiota and Drives Increased Escherichia coli Virulence and Drug Resistance in Young Adults
Source: Pathogens. 2022 Sep 18;11(9):1062. doi: 10.3390/pathogens11091062 (PMC9500718; doi:10.3390/pathogens11091062)
Supplement: Supplementary file 1 [file pathogens-11-01062-s001.zip › Table S1.pdf]

Table S1 Baseline characteristics of study participants

	Gender % (Prevalence)	Age (year) (mean ± SD)	BMI kg/cm^2 (mean ± SD)
Male	53.3% (160/300)	21.4 ± 0.7	20.8 ± 2.3
Female	46.7% (140/300)	21.9 ± 0.7	22.0 ± 2.9
