# Supplementary material for: Exposure to Veterinary Antibiotics via Food Chain Disrupts Gut Microbiota and Drives Increased Escherichia coli Virulence and Drug Resistance in Young Adults
Source: Pathogens. 2022 Sep 18;11(9):1062. doi: 10.3390/pathogens11091062 (PMC9500718; doi:10.3390/pathogens11091062)
Supplement: Supplementary file 1 [file pathogens-11-01062-s001.zip › Table S2.pdf]

Table S2: primers used in this study

Gene's name	Primer sequence (5' to 3')
aac(3)-IV	F: CTTCAGGATGGCAAGTTGGT<br>R: TCATCTCGTTCTCCGCTCAT
cat-A1	F: AGTTGCTCAATGTACCTATATAACC<br>R: TTGTAATTCATTAAGCATTCTGCC
cml-A	F: CCGCCACGGTGTTGTTGTTATC<br>R: CACCTTGCCTGCCCATCATTAG
qnr-A	F: AGAGGATTTCTCACGCCAGG<br>R: TGCCAGGCACAGATCTTGAC
qnr-B	F: GGMATHGAAATTCGCCACTG<br>R: TTTGCYGYYCGCCAGTCGAA
qnr-S	F: ACGACATTCGTCAACTGCAA<br>R: TAAATTGGCACCCTGTAGGC
tet-A	F: TTGGCATTCTGCATTCACTCG<br>R: CCACCCGTTCCACGTTGTT
tet-B	F: TTCACCGCATAGTCCCTT<br>R: TGCAATAAATCCGAGCAG
CTX-M	F: CGCTTTGCGATGTGCAG<br>R: ACCGCGATATCGTTGGT
CTX-M-1	F: GCTGTTGTTAGGAAGTGTGCCGC<br>R: GCCGCCGACGCTAATACATC
OXY	F: GGTTTTGGTAACTGTGACGGG<br>R: CAGAGTGCAGAGTGTTGCAG
TEM-1	F: ATAAAATTCTTGAAGACGAAA<br>R: GACAGTTACCAATGCTTAATC
*Mdt*B	F: TCTTCCCGGTACAGGACAAT<br>R: CATCAACGCCAACAAATGAG
*Mdt*F	F: CCGTACCGGTGGTTATTCTC<br>R: ATCGATTTATGCGTCGCTTC
*Mdt*G	F: CGGTATTGTCTTCAGCATTACATTTT<br>R: GGCGAGTCCACCCCAAA
*Mdt*L	F: TATCCCGCCGGGATTGATAT<br>R: CGCTTCGCTGGCATTGA
*iut*A	F: GGCTGGACATGGGAACTGG<br>R: CGTCGGGAACGGGTAGAATCG
*cva*C	F: CACACACAAACGGGAGCTGTT<br>R: CACACACAAACGGGAGCTGTT
iss	F: ATCACATAGGATTCTGCCG<br>R: ACAAAAAGTTCTATCGCTTCC
tsh	F: GGTGGTGCACTGGAGTGG<br>R: AGTCCAGCGTGATAGTGG
*pap*C	F: GACGGCTGTACTGCAGGGTGTGGCG

	R: ATATCCTTTCTGCAGGGATGCAATA
kps	F: GCGCATTTGCTGATCGTTG<br>R: CATCCAGACGATAAGCATGAGCA
Iha	F: CTGGCGGAGGCTCTGAGATCA<br>R: TCCTTAAGCTCCCGCGGCTGA
E. coli	F: TACCCCGAGGAAAAGAAATCAACC<br>R: CACACACTGATTCAGGCTCTGG
Taqman Probe	FAM 5´ CCCGTTCGCTCGCCGCTACTGG 3´ BHQ1
