# Supplementary figures and images for: Exposure to Veterinary Antibiotics via Food Chain Disrupts Gut Microbiota and Drives Increased Escherichia coli Virulence and Drug Resistance in Young Adults
Source: Pathogens. 2022 Sep 18;11(9):1062. doi: 10.3390/pathogens11091062 (PMC9500718; doi:10.3390/pathogens11091062)

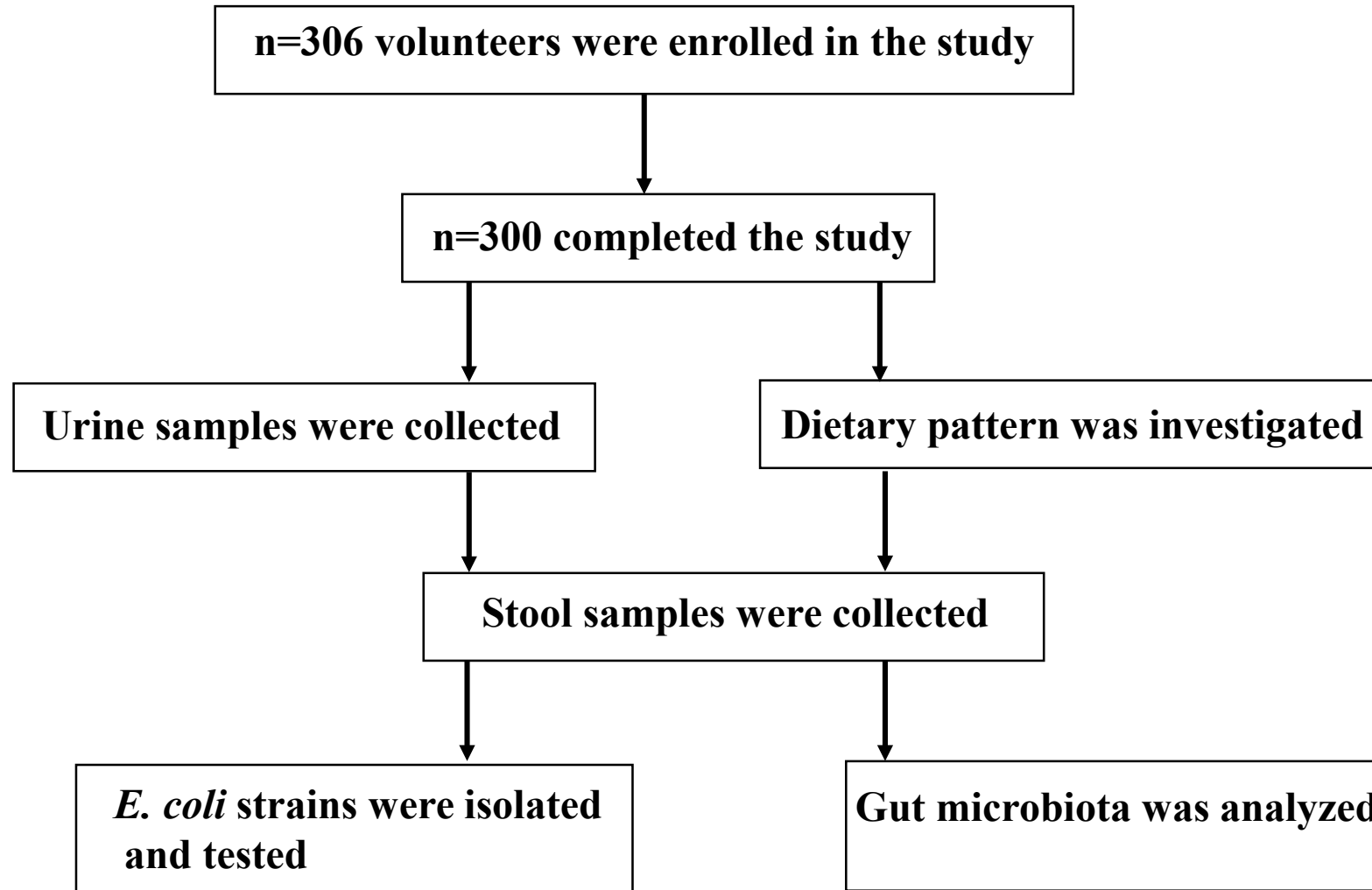

**Figure S1 The follow-up diagram of the present study**

Supplement: Supplementary file 1 [file pathogens-11-01062-s001.zip › Figure S1.pdf]

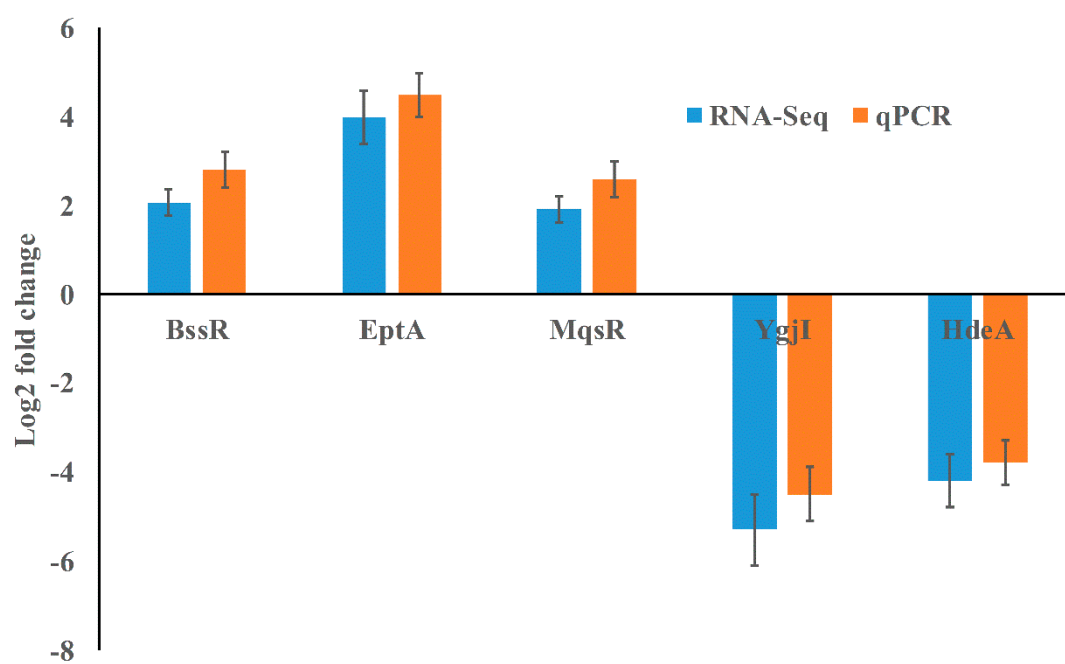

Figure S2: Comparison of qPCR result with RNA-Seq.

Supplement: Supplementary file 1 [file pathogens-11-01062-s001.zip › Figure S2.pdf]
